# Characterization of Destrins with Different Dextrose Equivalents 

**DOI:** 10.3390/molecules15085162

**Published:** 2010-07-29

**Authors:** Junliang Sun, Ruixiang Zhao, Jie Zeng, Guanglei Li, Xinhua Li

**Affiliations:** 1 School of Food Science, Henan Institute of Science and Technology, Xinxiang 453003, China; 2 College of Food Science, Shenyang Agricultural University, Shenyang 110161, China

**Keywords:** dextrin, characteristics, dextrose equivalent, viscosity, molecular weight, oligosaccharide

## Abstract

Dextrins are widely used for their functional properties and prepared by partial hydrolysis of starch using acid, enzymes, or combinations of both. The physiochemical properties of dextrins are dependent on their molecular distribution and oligosaccharide profiles. In this study, scanning electron microscopy (SEM), X-ray diffractometry (XRD), rapid viscoanalysis (RVA), high-performance Liquid Chromatography (HPLC) and gel permeation chromatography (GPC) were used to characterize dextrins prepared by common neutral and thermostable α-amylase hydrolysis. The dextrin granules displayed irregular surfaces and were badly damaged by the enzyme treatment. They displayed A-type X-ray diffraction patterns with a decrease of intensity of the characteristic diffraction peaks. The RVA profiles showed that the viscosity of dextrin decreased with the increase of its Dextrose Equivalent (DE) value. According to HPLC analysis, the molecular weight, degree of polymerization and the composition of oligosaccharides in dextrins were different.

## 1. Introduction

Native starch granules are semi-crystalline and can resist hydrolysis by amylases. When gelatinized, they are readily hydrolyzed and converted to sugars and dextrins, thereby reducing the molecular weight of starch molecules (amylose and amylopectin) [1,2]. Dextrins are starch hydrolysis products produced by acid hydrolysis, enzyme hydrolysis, or a combination of both [1,3]. Dextrin is one of several carbohydrates having the same general formula as starch, but dextrin and starch are structurally different as dextrin is a smaller and less complex molecule [4]. 

The extent of hydrolysis is normally expressed in terms of the ‘‘dextrose equivalent’’(DE), a quantity usually determined by titration and a measure of the total reducing power of the sugars present relative to a dextrose (D-glucose) standard, on a dry mass basis. The DE value is inversely related to molecular weight, *i.e.*, the degree of polymerization (DP), and is an indicator of the degree of hydrolysis. Thus, glucose has a DE value of 100, while intact starch maybe have an effective DE of zero [5]. Starch hydrolyzates with DE values below 20 are referred to as maltodextrins. Dextrins with the same DE can have different properties and molecular compositions, depending on the starch and how it is digested [6] and this may greatly affect the properties of dextrins such as hygroscopicity, fermentability, viscosity, sweetness, stability, gelation, solubility and bioavailability and so on. 

Though partial hydrolysis of starch has traditionally been carried out by using acids, acid hydrolysis is being replaced by enzymatic hydrolysis for the production of maltodextrins [6]. The most widely used enzymes for production of maltodextrins using partial hydrolysis of starch are α-amylase from *Bacillus* sp. [7,8,9,10,11]. Enzyme hydrolysis with α-amylase efficiently hydrolyzes the α-(1→4) linkages, but not the α-(1→6) linkages, leaving behind a small amount of high-molecular-massnresidues [1].

More information is available about starch structure and properties, but there is less information on dextrins. As has been stated, dextrins with different DE values can have different properties and hence different functionality in a particular desired application. Thus, a complete oligosaccharide profile, in addition to DE, is desirable in order to better understand a dextrin’s physical and biological functionality. This paper focuses on the characteristics of dextrins with different DE by the use of scanning electron microscopy (SEM), X-ray diffractometry (XRD), rapid viscoanalysis (RVA), gel permeation chromatography (GPC) and high-performance liquid chromatography (HPLC). 

## 2. Results and Discussion

### 2.1. SEM of dextrins with different DE values

**Figure 1:**
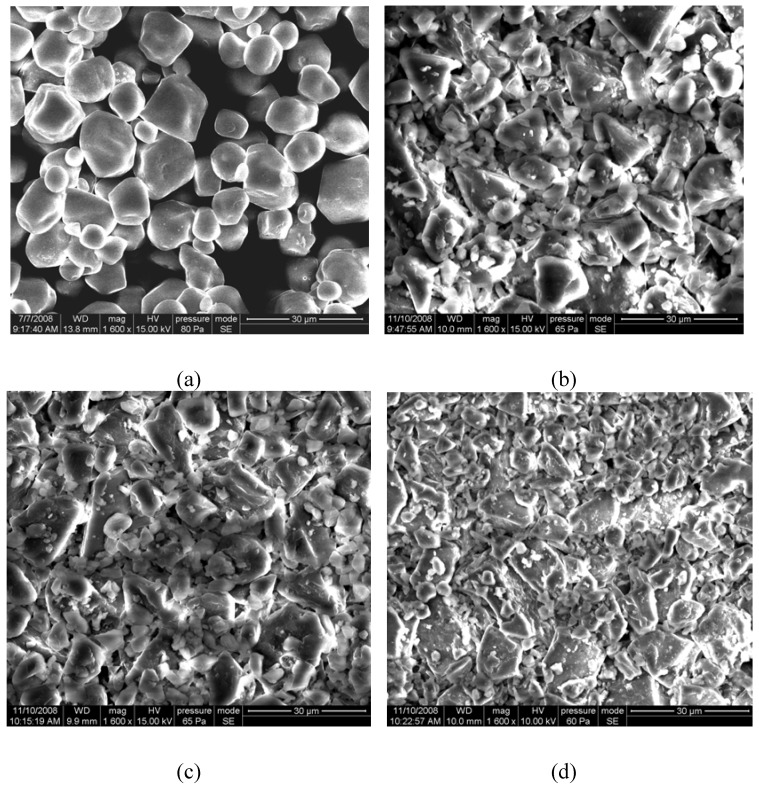
SEM photographs of the starch and the dextrins: (a) native starch; (b) dextrin (DE 9); (c) dextrin (DE 15); (d) dextrin (DE 22).

The dextrin granules were comparable in size when viewed by a SEM instrument. SEM photographs of the native starch and the dextrins with DE 9, DE 15 and DE 22 are displayed in Figure 1. The shape and size of the obtained dextrin granules were significantly different, compared with that of the native starch. The native starch granules appeared oval, elliptic or spherical (Figure 1a, and no fissures were noticed on the surface of the starch granules. When the starch granules were subjected to enzymatic hydrolysis, no intact starch granules could be found and all the fragments were conglutinated together due to the heavy enzyme erosion. The dextrin granules were irregular on the surface, indicating that most of the starch granules were badly damaged by the enzyme treatment (Figure 1b,c,d). The above results showed that the core part of the starch granule (the amorphous regions) was more easily attacked by the enzymes and as a result, the core was completely decomposed. Then the enzyme molecules began to attack the crystalline regions of the starch granules, so only the fractured surface could be found in the SEM photographs of the dextrins.

### 2.2. X-ray study of dextrins

**Figure 2:**
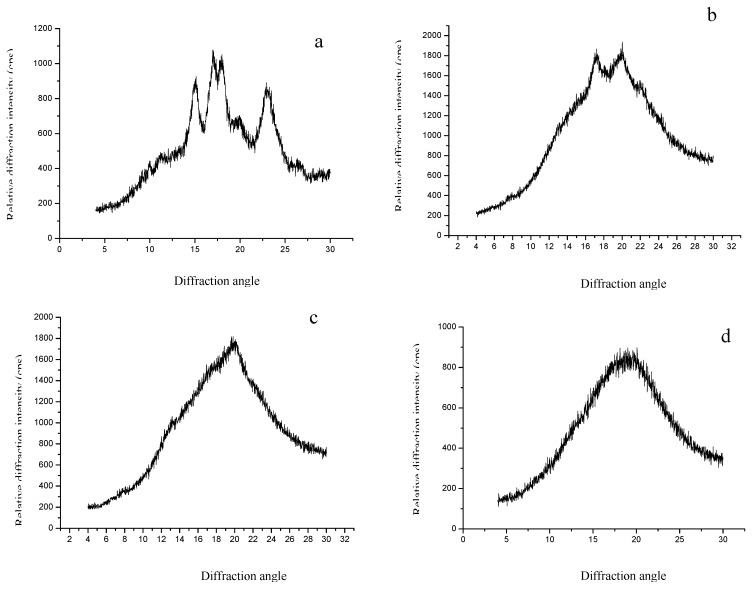
X-ray diffraction spectra of the starch and the dextrins: (a) native starch; (b) dextrin (DE 9); (c) dextrin (DE 15); (d) dextrin (DE 22).

The X-ray diffraction patterns of the corn starches and the dextrins are shown in Figure 2. The starch powders showed the strongest diffraction peak at 2θ values of 14.97°, 17.03°, 17.90° and 22.86° (Figure 2a), which indicated that the crystal type of the starch was a characteristic A-type [12,13]. After hydrolysis by α-amylases, compared with native starch the intensity of the characteristic diffraction peaks of the dextrins decreased, and some peaks had been combined (Figure 2b,c), or even disappeared (Figure 2d). But the A-type crystal type was partly preserved. 

### 2.3. Viscosity of diluted solutions

**Figure 3:**
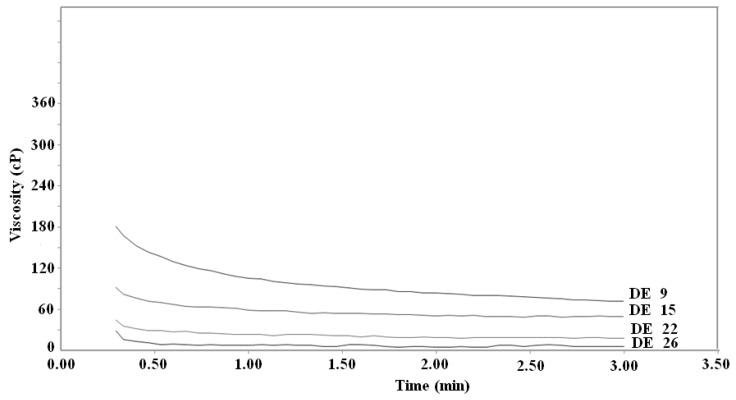
The dependence of viscosity on DE of various dextrins.

Viscosity data for commercial dextrins are normally available from their manufacturers. In this study, the viscosity of dextrin solutions with the concentration 10% (w/v) was determined at 30 ºC. The apparent viscosity values obtained are shown in Figure 3. The viscosity of dextrin decreased with the increase of DE value. For the same sample, the viscosity decreased gradually during stirring with a 160 rpm rotation speed, but the viscosities were basically stable after stirring 2.5 min and the values were 88, 54, 21 and 7cP for DE 9, 15, 22 and 26, respectively. When the DE value of dextrin was higher than 22, the viscosity was similar to that of distilled water. 

It was reported that the viscosity of commercial dextrins increased approximately linearly or curvilinearly with both the DP and the concentration of the saccharide [6], but that the relationship between viscosity and DE was linear [5]. Degradation of starch has been considered responsible for the viscosity changes caused by enzyme hydrolysis [4]. This provided a method to determine the DE value by testing viscosity.

### 2.4. Gel permeation chromatography

**Figure 4:**
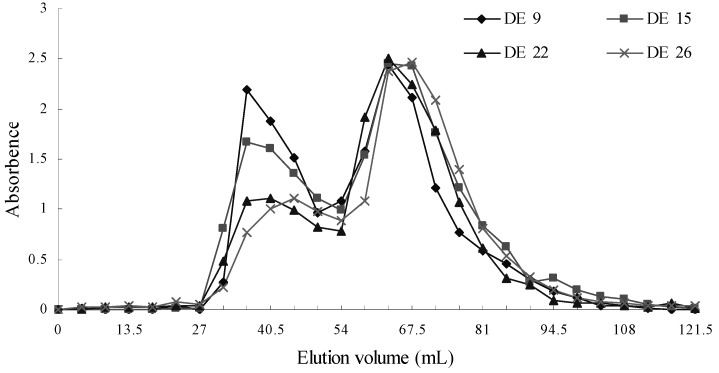
Elution profile on sephadex G 50 column of starch and dextrin.

Figure 4 shows the GPC profiles of different dextrins. All samples showed a smaller peak at the exclusion limit of the columns named fraction I, followed by much larger amounts of lower molecular weight compositions named fraction II. The elution profiles of all dextrins were bimodal distributions. The area of fraction I decreased with the increase of DE value, but the area of fraction II went up. The proportions of fractions I or II were 90:100, 68:100,45:100 and 45:100 for the dextrins with DE value 9, 15, 22 and 26, respectively. These results indicated that the proportion of large molecules gradually decreased during enzymatic hydrolysis.

### 2.5. Molecular weight of dextrins

**Figure 5:**
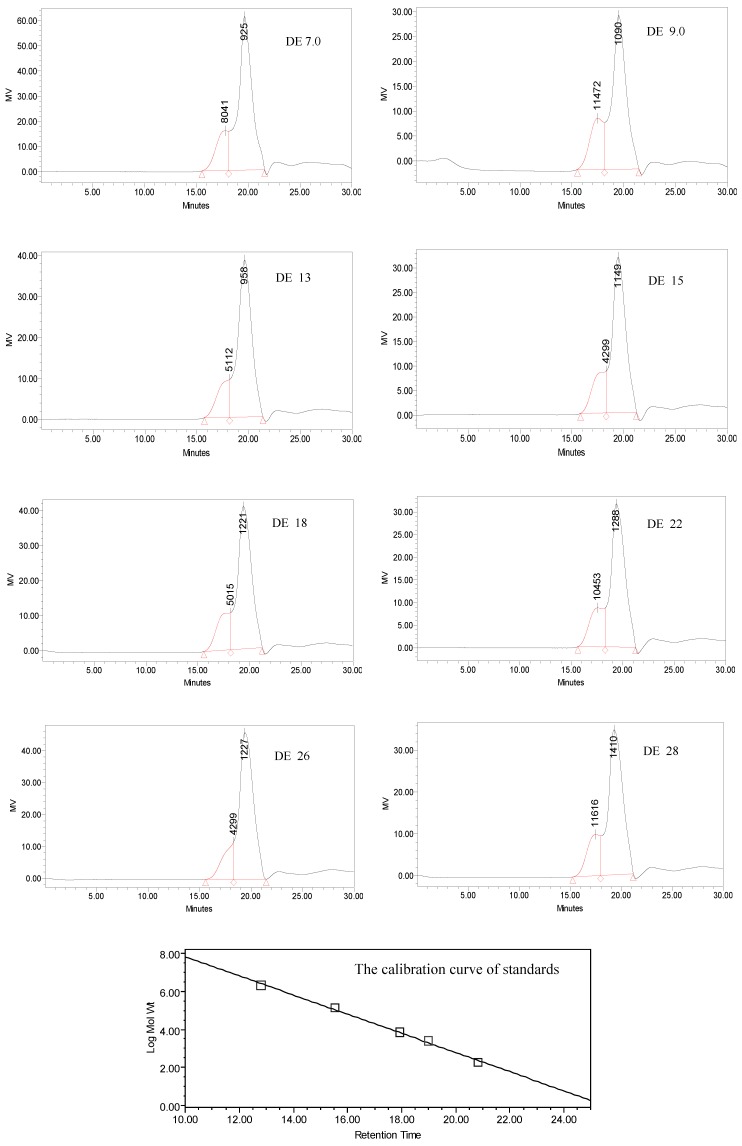
Molecular weight distribution of dextrins with different DE value.

Molecular weights of dextrins with different DE value were determined by HPLC. Figure 5 shows the resulting molecular weight distribution. From Figure 5, all dextrins displayed a bimodal distribution. These results were consistent with those obtained from gel permeation chromatography. Table 1 summarizes the results with the number of peaks quantitated for each dextrin sample, along with the retention time, peak area, number average molecular weight (Mn), weight average molecular weight (Mw), molecular weight at peak (Mp), and average degree of polymerization (DP). From Table 1 it could be seen that as DE value increased the peak area, Mn, Mw, MP, and DP of polymers detected by HPLC changed irregularly. In brief, the molecular weight of Peak 1 (peak on the left) was larger than that of Peak 2 (peak on the right) for all dextrin samples. The largest area of Peak 1 corresponded to the dextrin with DE 9 and the smallest one was that of the dextrin with DE 26. The largest DPn of Peak 1 was of the dextrin with DE 28 and the smallest one was of the dextrin with DE 26. The DPn of Peak 1 in all dextrin samples were lower than 86 and the DPn of Peak 2 was not higher than 6 for all dextrins. The smallest DPn of Peak 2 was that of the dextrin with DE 7. The DPn of dextrins in this study were far lower than that of the previous reports. Liu *et al*. compared the molecular weight of two kinds of dextrins with DE values of 11.4 and 11.9, and found that their DPn were 152.2 and 133.7, respectively [14]. These results indicated that the molecular weight distribution of dextrins prepared by common neutral α-amylase and thermostable α-amylase in this study was more even. 

**Table 1:** Molecular weight of dextrins with different DE values.

Name	DE value	Retention Time (min)	Area	Area (%)	Mn	Mw	Mp	DPn	DPw
Peak 1	7	17.774	1297303	18.69	11132	15761	8041	68.7	97.3
	9	17.467	934333	24.06	11751	17174	11472	72.5	106.0
	13	18.167	733533	16.84	10485	14810	5112	64.7	91.4
	15	18.317	757873	20.91	9290	13403	4299	57.3	82.7
	18	18.183	966087	20.19	10881	15825	5015	67.2	97.7
	22	17.547	870223	22.66	10346	15785	10453	63.9	97.4
	26	18.317	831988	15.98	8748	12776	4299	54.0	78.9
	28	17.456	876181	19.97	13824	19990	11616	85.3	123.4
Peak 2	7	19.647	5644866	81.31	633	1222	925	3.9	7.5
	9	19.505	2948976	75.94	760	1383	1090	4.7	8.5
	13	19.616	3621699	83.16	732	1262	958	4.5	7.8
	15	19.459	2865852	79.09	788	1260	1149	4.9	7.8
	18	19.407	3817883	79.81	806	1344	1221	5.0	8.3
	22	19.360	2970766	77.34	783	1286	1288	4.8	7.9
	26	19.403	4375426	84.02	763	1248	1227	4.7	7.7
	28	19.282	3511781	80.03	876	1569	1410	5.4	9.7

Mn means number average molecular weight; Mw means weight average molecular weight; Mp means molecular weight at peak; DPn=Mn/162, DPw =Mw/162.

### 2.6. Oligosaccharide composition of dextrins

**Figure 6:**
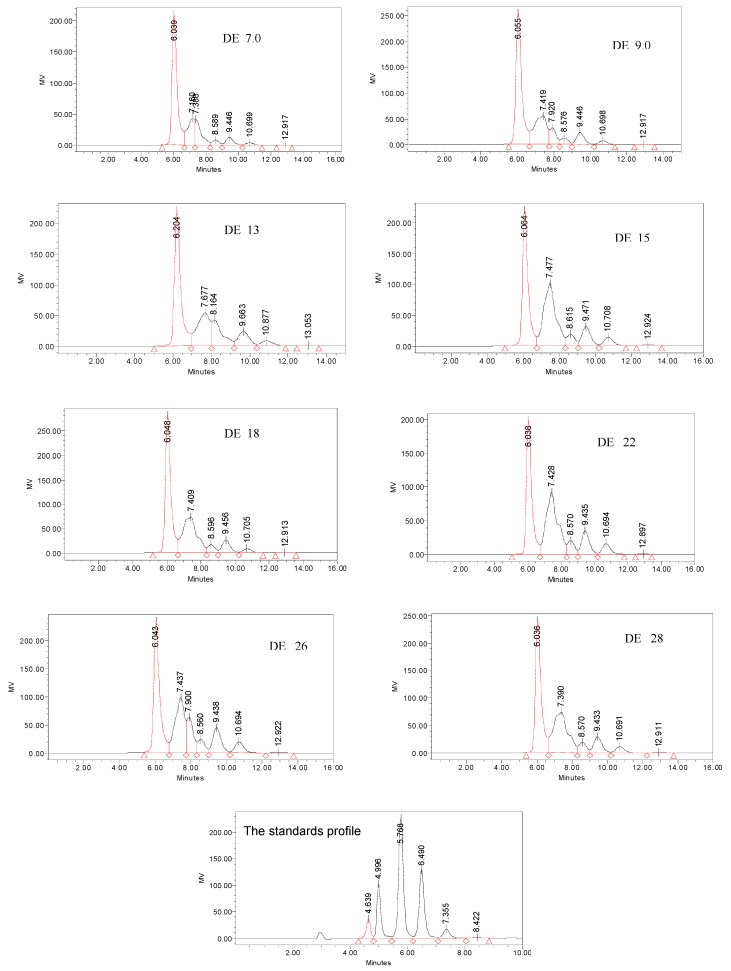
HPLC oligosaccharide profile of dextrin with different DE value. Number in the figurerepresented retention time of glucose and oligosaccharides.

As mentioned above, the DPn of Peak 2 in all dextrins were not higher than 6, but the area of Peak 2 were above 75. This means that there were lots of oligosaccharides with DP < 6 in the dextrins. Figure 6 illustrates the differences of oligosaccharides in HPLC profiles of dextrins with different DE values. The results were summarized in Table 2 with the area percent of peaks quantitated for oligosaccharides. From Table 2 it could be seen that there were great differences for the content malto-oligosaccharides with DP 4-6 in different dextrins. According to the retention time of standard oligosaccharides, the proportion of maltotriose and maltotetrose (G3+G4) was the largest in all dextrin samples, and the proportion of maltopentaose (G5) was higher than that of maltohexaose (G6) and other oligosaccharides with DP ≥ 7 except for the dextrin of DE 22. There were no glucose and maltose in any of the dextrin samples. 

**Table 2:** Proportion of sugar of dextrins with different DE value.

DE value	Area(% )			
	G3 + G4	G5	G6	DP≥ 7
7	59.92	30.65	2.97	6.47
9	54.91	32.81	3.29	8.99
13	48.82	23.80	15.19	12.19
15	41.48	40.55	4.76	13.21
18	51.58	34.77	4.03	0.62
22	39.8	40.05	5.31	14.84
26	40.66	38.56	4.84	15.93
28	47.15	36.28	4.79	11.78

G3-G6 are malto-oligosaccharides with DP 3-6, respectively.

## 3. Experimental

### 3.1. Materials and reagents

Commercial corn starch was purchased from Changzhi Jinze Biological Engineering Co. Ltd (Zhengzhou, China). The common neutral α-amylase (2000 U/mL) and thermostable α-amylase (20000 U/mL) were from Zhengzhou Fuyuan Bio-products Co. Ltd ( Zhengzhou, China). Sephadex G 50 and standards with molecular weight 2,000,000, 133,800, 41,100, 21,400, 4,600, 180 were products of Sigma. Acetonitrile was of chromatographic grade. Other reagents were of analytical grade. 

### 3.2. Preparation of Dextrin

Corn starch (50 g) was mixed with distilled water (150 mL) and stirred for 10 min. This solution was mixed with 0.1 mol/L NaOH to pH 6.0 and 0.1 % (w/v) CaCl_2_, successively. Then, common neutral α-amylase and thermostable α-amylase were added into the above solution to hydrolyze the starch. After enzyme denaturing by high pressure (Shanghai Tocan Science and Technology Co. Ltd) and cooling, the DE-value of the product was determined. The dextrins with different DE values were obtained under the following conditions: reaction temperature, 90 ºC; hydrolysis time, 3, 6, 9, 12, 15, 18, 21, 24 min (giving dextrins with DE 7, 9, 13, 15, 18, 22, 26 and 28, respectively); addition of common neutral α-amylase, 4 U/g starch; and addition of thermostable α-amylase, 7 U/g starch. Obtained products were centrifuged, freeze-dried and used to property evaluation.

### 3.3. Determination of dextrin’s DE-value

The sample (1 g) was diluted to 50 mL and filtered. Then the above solution (0.5 mL) was mixed with 3,5-dinitrosalicylic acid to measure the content of reducing sugar with glucose as standard. DE value was calculated in the following formula:

DE-value = reducing sugar content (glucose) / total solids content × 100%

### 3.4. Scanning electron micrographs of dextrin

Scanning electron microscopy (SEM) micrographs was recorded with a Quanta 200 environmental scanning electron microscope (FEI Company, USA). The samples were evenly distributed on SEM specimen stubs with double adhesive tape. The micrographs were obtained with an accelerating potential of 15 kV under low vacuum. The micrographs obtained were used to detect any damage to the starch granules.

### 3.5. X-ray diffractometry

Monochromatic Cu Ka radiation (wavelength = 1.54056 Å) was produced by a D/MAX 2500V/ PC X-ray diffractometer (Rigaku Americas Corporation, Japan). The samples were incubated in a chamber at 100% RH for 24 h and then packed tightly in a rectangular aluminum cell. The samples were exposed to the X-ray beam from an X-ray generator running at 36 kV and 20 mA. The scanning regions of the diffraction angle, 2θ, were 0–30º, which covered most of the significant diffraction peaks of the starch crystallites. Other operation conditions included: step interval 0.02, scan rate 4º/min, Sollet and divergence slit 1º, receiving slit 1º, and scattering slit 0.16º. Duplicate measurements were made at ambient temperature. Radiation was detected with a proportional detector.

### 3.6. Viscosity of dextrins

The measurements of viscosity of the diluted dextrin solutions (10%,w/v) were evaluated with a Rapid Viscoanalyzer (RAV-4, Newport Scientific, Warriewood, Australia). Measurements were made at 30 ºC for 3 min, Speed 160 rpm. 

### 3.7. Gel permeation chromatography

Gel permeation chromatography (GPC) profiles were obtained according to the method described by Manhanta and Bhattacharya [15] with some modification. An aliquot of distilled water (10 mL) was added to a dextrin sample (0.1 g). The sample solution, purged with nitrogen, was heated for 3 to 4 min in a boiling water bath, and then cooled to 25 ºC. The solution was loaded into a Sephadex G 50 column (diameter 1.0 cm × height 70 cm) and eluted at a rate of 18 mL/h (4.5 mL of effluent were collected in every tube) with 50 mmol/L sodium chloride. Then 1 mL of the efluent in each tube was used to analyze the carbohydrate content and elution volume response at λ = 620 nm by the phenol-sulfuric acid method.

### 3.8. Molecular weight determination of dextrins

HPLC was performed with a Waters 600 chromatograph (Waters, USA) equipped with a 2401 refractive index detector. The column used was a Ultrahydrogel™Linear (300 mm × 7.8 mm id × 2). The mobile phase consisted of 0.1 M NaNO_3_. The column temperature was 45 ºC. The flow-rate was 0.9 mL/min and the injection volume was 10 μL. The molecular weight calibration curve was standardized by standards with molecular weight 2,000,000, 133,800, 41,100, 21,400, 4,600 and 180. 

### 3.9. Composition determination of dextrins with different DE value

Before testing the oligosaccharide composition of dextrins, the samples had been treated by desalination and micro-filtration membrane. HPLC was performed with a Waters 600 chromatograph (Waters, USA) equipped with a 2401 refractive index detector and model M730 Data Processor. The column used was a Sugarpak1 with water as mobile phase and the column temperature was 85 ºC. The flow rate was 0.4 mL/min and the injection volume was 10 μL.

## 4. Conclusions

Scanning electron microscopy showed that dextrin granules were irregular on the surface and most of the starch granules were badly damaged by enzyme treatment. X-ray analysis showed that the intensity of the characteristic diffraction peaks of the dextrins decreased, as perhaps the crystalline regions were partially hydrolyzed by the enzymes or partially gelatinized. The RVA profiles indicated that the viscosity of dextrin decreased with the increase of DE value. Molecular distribution (molecular weight, degree of polymerization *etc*.) and the composition of oligosaccharides in dextrins with different DE value were greatly different. 
